# Validation of the eating pathology symptoms inventory (EPSI) in Swedish adolescents

**DOI:** 10.1186/s40337-024-01027-7

**Published:** 2024-05-27

**Authors:** Andreas Birgegård, Rasmus Isomaa, Elin Monell, Johan Bjureberg

**Affiliations:** 1 Department of Medical Epidemiology and Biostatistics, Karolinska Institutet, Stockholm, Sweden; 2 Wellbeing Services County of Ostrobothnia, Vasa, Finland; 3 Faculty of Education and Welfare Studies, Åbo Akademi University, Vasa, Finland; 4 Centre for Psychiatry Research, Department of Clinical Neuroscience, Karolinska Institutet, & Stockholm Health Care Services, Region Stockholm, Stockholm, Sweden

**Keywords:** Eating disorder, Questionnaire, Factor analysis, Validity, Gender

## Abstract

**Background:**

Eating disorders (ED) are associated with symptoms across body image, disordered eating, and exercise-related domains, and while predominantly affecting females, ED in males is also a significant concern. However, popular self-report methods insufficiently capture male presentations. This study aimed (1) to validate the first Swedish translation of the Eating Pathology Symptoms Inventory (EPSI), which was designed to overcome limitations in previous measures, and (2) compare genders gender-specific manifestations of eating pathology, depression, and anxiety in Swedish high-school students.

**Methods:**

Participants were 359 high-school students (47% males) aged 17.0 years (range 15–21).

**Results:**

Confirmatory factor analysis and correlation patterns showed support for the 8-factor structure and convergent validity, but poorer discriminant validity may suggest caution in interpreting single scales as evidence of ED pathology. Gender comparisons were broadly consistent with previous research.

**Conlusions:**

: The Swedish EPSI may be used to asses ED symptoms, but caution is suggested in interpreting some scales in isolation as indicative of ED pathology.

**Supplementary Information:**

The online version contains supplementary material available at 10.1186/s40337-024-01027-7.

## Plan English summary

Indivudals with eating disorders (ED) experience symptoms that have to do with body image, disordered eating, and physical exercise. Most who get an ED are female, but males are also affected. However, many symptom questionnaires do not capture how males experience their illness very well. In this study, we wanted to (1) test a Swedish translation of the Eating Pathology Symptoms Inventory (EPSI), which was designed to overcome some limitations in other questionnaires, and (2) compare male and female Swedish high-school students on ED symptoms, depression, and anxiety. Participants were 359 students (47% males) aged ≈ 17 years (range 15–21). Statistical analysis showed that the 8 suggested scales of the EPSI overall function as expected, and that the EPSI seems to measure ED symptoms well, but that it also has some overlap with depressive and anxiety symptoms. Males and females differed from one another in ways that resembled what other researchers have found. We conclude that the Swedish EPSI works well, but that some of the scales might not, on their own, suggest that a person has problems with ED, unless other more ED-specific scales also indicate such symptoms.

**Supplementary Information:**

The online version contains supplementary material available at 10.1186/s40337-024-01027-7.

## Introduction

Eating disorders (ED) are manifested in a broad pattern of disturbances across body image, disordered eating, and exercise-related domains. A recent review found a lifetime prevalence of 2.58–8.4% for young women and 0.74–2.2% for young men [1]. Only a minority seek help for their ED however, and males are significantly less likely to seek help, be diagnosed, and recieive treatment than females [2, 3], underscoring the need for assessment methods that capture ED pathology equally well in both genders. EDs in women, in general, tend to revolve around thinness-oriented behaviours and cognitions, such as restricted eating, fear of becoming fat, and compensatory measures aimed at controlling energy intake/uptake and burning calories [3, 4]. In men, ED pathology has been suggested to be muscularity-oriented rather than thinness-oriented [3, 4]. In both manifestations, body dissatisfaction and overvaluation of body shape and weight are core features of the pathology, and the different orientations between muscularity/thinness are rather reflected in the overt pathological behaviours, such as wanting to gain lean muscle weight vs. losing weight to attain thinness [5, 6]. Although conclusions are still tentative given that less than 1% of ED research has focused on men [7], such gender differences are in line with sociocultural body ideals for men and women [8]. The muscularity and thinness orientations in symptomatology are not to be seen as mutually exclusive, but rather trends reflecting that EDs manifest in more than one traditional, gender-specific way.

Self-report measures are widely used in both ED research and clinical work. Available measures, with the Eating Disorder Examination Questionnaire (EDE-Q; [9, 10]) being the most common, have limitations including inconsistent factor structures, bias toward ED presentations in females, poor discriminant validity, and reduced psychometric properties in specific populations [11–15]. To overcome these limitations, the 45-item Eating Pathology Symptoms Inventory (EPSI; [11]) was developed. The EPSI was intended to encompass several important dimensions of ED pathology, some of which are not included in previously developed instruments, to differentiate specific ED behaviours, and assess dietary restraint better than currently available measures. The suggested eight factors of the EPSI are Body Dissatisfaction, Binge Eating, Cognitive Restraint, Excessive Exercise, Restricting, Purging, Muscle Building, and Negative Attitudes toward Obesity. Of these, Body Dissatisfaction appears to represent a broader and more general measure of eating pathology [11]. The eight-factor structure has since been replicated with good model fit in another American college sample [6], in a Chinese version in a Chinese speaking sample in the U.S [5]. , a Farsi version in Iranian adolescents and university students [16], and in an international sample of sexual minority men [17]. Further, the factor structure has been confirmed in adolescents, and measurement invariance has been shown between adolescents and adults [16, 18]. However, Coniglio and colleagues found only limited support for the factor structure [19] and the authors suggested scale heterogeneity (more than one construct in one scale, especially regarding Purging and Excessive Exercise) as a possible reason for these shortcomings. Tests of measurement invariance have generally concluded that the EPSI can be used also across gender and body weight groups [11, 16, 18].

The EPSI has more broadly demonstrated gender differences in eating pathology. Among college students, women scored higher on more traditional ED dimensions such as Body Dissatisfaction, Binge Eating, Cognitive Restraint, Purging, and Restricting, while men scored higher on Negative Attitudes toward Obesity and Muscle Building [11]. The same gender pattern was visible also in another college sample [6] and in the above-mentioned Chinese speaking sample [5]. In a comparison of gender differences among general psychiatric patients, women scored higher on Body Dissatisfaction, Cognitive Restraint and Purging, and men scored higher on Excessive Exercise and Muscle Building [11]. Indeed, a seven-factor solution of the EPSI, omitting the Muscle Building factor, has been shown to produce an excellent fit in women [6].

The aim of the present study was two-fold. First, we aimed to validate a Swedish version of the EPSI, to enable further research using this measure as well as its possible clinical use given the shortcomings of other commonly used instruments. This was done by assessing its factor structure and convergent and discriminant validity via gender-separate correlations with cognitive ED symptoms, and symptoms of depression, anxiety, and obsessions/compulsions. ED symptoms are however substantially associated with symptom domains such as anxiety and depression [20], and to demonstrate discriminant validity therefore, the EPSI should demonstrate statistically significantly stronger correlation to ED symptoms than to general psychiatric symptoms. Second, we aimed to study gender-specific manifestations of eating pathology in Swedish high-school students using the EPSI and the EDE-Q, as well as comparing genders on depression and anxiety to complement gender-specific validity analyses and enable evaluation of the full pattern of gender differences.

## Method

### Participants

Participants were 359 convenience-sampled high school students (males = 169/47%, females = 190/53%). Out of a total of 835 students who were given the opportunity to participate, 388 provided data (46% response rate), whereof 29 with no EPSI data were removed prior to analysis. Mean age was 17.0 (*SD* = 1.04, range 15–21) with a small significant sex difference (males = 16.9 [*SD* = 1.00], females = 17.1 [1.03], *t*=-2.071, *p* = .039; Cohen’s *d* = 0.22).

### Instruments

The EPSI ([11]; rated 0 = Never to 4 = Very often), described above, measures the last four weeks. Cronbach’s alpha for the full sample was acceptable to good for all scales in the current sample (Table 1). The Swedish version was created for the study by translation to Swedish by two native Swedish speakers fluent in English who were experienced ED researchers, back translation by a native English speaker (also an experienced ED researcher and clinician) fluent in Swedish, and any discrepancies resolved by discussion between these three until consensus was reached.^1^

**Table 1:** Cronbach’s alpha for the EPSI, RCADS, and EDE-Q, descriptive statistics per gender, and multivariate gender comparisons with age as covariate including effect size (partial eta squared; η_*p*_^*2*^). Significantly higher group means (SD) are **bold** for ease of interpretation

Measure	Scale	α	Female M (SD)	Male M (SD)	F	*p*	η_*p*_^2^
EPSI^1^	Body Dissatisfaction	0.87	**13.4 (6.68)**	6.6 (5.56)	105.070	< 0.001	0.228
	Binge Eating	0.84	9.4 (6.78)	7.8 (5.49)	4.251	0.022	0.012
	Cognitive Restraint	0.76	**4.1 (3.10)**	2.5 (2.38)	25.933	< 0.001	0.068
	Purging	0.87	1.9 (3.50)	1.5 (3.75)	0.829	0.346	0.002
	Restricting	0.86	**7.0 (5.62)**	4.2 (4.50)	23.164	< 0.001	0.066
	Excessive Exercise	0.84	6.3 (5.03)	7.3 (5.33)	3.202	0.070	0.009
	Negative Attitudes toward Obesity	0.86	3.6 (4.06)	**6.2** (4.89)	30.812	< 0.001	0.080
	Muscle Building^1^	0.72	2.8 (2.97)	**4.4** (3.67)	26.383	< 0.001	0.069
EDE-Q^2^	Global score	0.87	**2.0 (1.46)**	0.9 (0.87)	72.384	< 0.001	0.174
RCADS^1^	Social phobia	0.88	**14.6 (5.92)**	9.58 (5.46)	68.382	< 0.001	0.165
	Panic	0.89	**8.7 (5.74)**	4.4 (4.41)	54.968	< 0.001	0.137
	Major depression	0.85	**11.9 (5.26)**	7.9 (4.92)	51.393	< 0.001	0.129
	Separation anxiety	0.82	**4.9 (3.99)**	2.3 (3.27)	40.350	< 0.001	0.104
	Generalized anxiety	0.83	**8.1 (3.80)**	5.0 (3.46)	56.588	< 0.001	0.145
	Obsessive-Compulsive	0.75	3.9 (3.20)	3.9 (3.48)	0.045	0.832	0.000

Note. EPSI = Eating Pathology Symptoms Inventory; RCADS = Revised Children’s Anxiety and Depression Scale; EDE-Q = Eating Disorders Examination Questionnaire

^1^ Female *n* = 190, Male *n* = 169; ^2^ Female *n* = 186, Male *n* = 165

The Revised Child Anxiety and Depression Scale (RCADS; [21]) is a 47-item (rated 0 = Never to 4 = Always) measure of anxiety and depression in 8 to 18 year old children/adolescents, with no specified time frame. Subscales measure symptoms of separation anxiety, social phobia, generalized anxiety, panic, obsession-compulsivity, and depression. Good internal consistency and subscale- and construct validity have been reported [22]. Gender differences were investigated for all subscales in the present study, but only the Total Internalizing scale for validity analyses, computed as the sum of all six subscales. Cronbach’s alpha for the full sample was acceptable to good for all subscales (Table 1).

The EDE-Q version 6.0 [10], with 28 items (rated 0 = No days to 6 = Every day) and a four-week time frame, is the most commonly used self-report questionnaire for ED symptoms, consisting of four purported subscales (Restraint, Eating Concern, Shape Concern, and Weight Concern) whose mean is a Global Score, as well as ratings of presence and frequency of key symptom behaviors (binge eating, purging, and excessive exercise). Since the intended factor structure has not been replicated, only the Global scale will be used in the present study; other acceptable psychometric properties have however been reported, including internal consistency and validity [23]. Cronbach’s alpha for the full sample was good (Table 1).

### Procedure

We contacted 51 high schools (Swedish ”gymnasium”) by letter to the principal: 26 declined due to lack of time, 20 did not respond, and five schools in two cities in the mid-region of Sweden decided to participate. At three schools, the principals only distributed the questionnaire link to social science students, who completed them in the classroom. The remaining two schools distributed the questionnaire link to all students via email, and they were completed at a place and time of their choosing. The six questionnaires (three not included in the present study) were completed using the online secure BASS system hosted by Karolinska Institute. Following questions about gender (male/female/other) and birth date, the questionnaire order was randomized for each participant to avoid systematic order/priming effects. Since the online interface did not allow skipping questions, there were no missing data. Student health teams were contacted at each school to alert them to the possibility that students might approach them with concerns related to the questionnaires, and contact information to the principal investigator was available to them and to each participant. Informed consent was collected from the students (not parents) in accordance with Swedish law. Participants were sent an electronic gift certificate (if they provided a phone number) worth approx USD 10 on completion of the survey. The study was approved by the Regional Ethical Review Board in Stockholm (#2018/11–31/1).

### Statistical analysis

#### Factor structure

Confirmatory factor analysis (CFA) was used to determine how well the original eight-factor model and the seven-factor model (excluding Muscle Building) fit the data. Models were fitted using Mplus 8.0 and weighted least square mean and variance adjusted (WLSMV) estimator [24]. We examined several goodness-of-fit indices, following recommendations: the chi square test, the Comparative Fit Index (CFI), the Standardized Root Mean square Residual (SRMR), and the Root Mean Square Error of Approximation (RMSEA) with 90% confidence intervals (CI: s). The following guidelines were used [25, 26]: good fit was determined based on a combination of CFI > 0.95, SRMR < 0.08, and RMSEA < 0.06, and acceptable fit was CFI > 0.90 and RMSEA < 0.08.

#### Convergent and discriminant validity

Pearson correlations between EPSI scales and EDE-Q Global score were computed, separated by gender, and male vs. female correlations were compared by *z* test with 2-sided *p*-values [27]. Convergent and discriminant validity were assessed, separated by gender, by examining whether correlations between the EPSI scales and the EDE-Q Global score were significantly (*p* < .05) different from correlations with the RCADS Total Internalizing scale (with the RCADS-EDE-Q correlation as reference), tested using *z* test with 2-sided *p*-values [28].

#### Mean gender differences

We performed multivariate generalized linear model (GLM) analyses of the EPSI and RCADS subscales, and univariate GLM for the EDE-Q Global scale, using age as covariate due to females being slightly but statistically significantly older. Effect sizes were calculated as partial eta squared (*η*_*p*_^*2*^; small ≥ 0.01, moderate ≥ 0.06, and large ≥ 14). Due to the many comparisons, we used alpha < 0.001 for these analyses.

## Results

### Factor structure

The eight-factor correlated traits model based on all 45 items of the EPSI showed acceptable fit (χ^2^(917) = 2046.465, *p* < .001; RMSEA = 0.059 [90% CI 0.055, 0.062]; CFI = 0.921; SRMR = 0.088). Factor loadings were all significant in the model. All correlations between latent factors, except for Muscle Building and Body Dissatisfaction (*r* = .099, *p* = .114), were statistically significant and in the range 0.151 − 0.713. The 7-factor correlated traits model based on 40 items, excluding items belonging to the Muscle Building scale, evidenced good fit (χ^2^(717) = 1400.167, *p* < .001; RMSEA = 0.052 [90% CI 0.048, 0.056]; CFI = 0.947; SRMR = 0.075). All items had significant factor loadings and significant correlations between latent factors ranging from 0.110 to 0.729. In within-EPSI correlations separated by gender, Muscle Building correlated notably more strongly with other symptoms in males than in females (see Supplementary material, Table S1).

### Convergent validity

The EPSI was in general significantly correlated with the EDE-Q in both genders (Table 2). The EPSI scales measuring more traditional ED pathology showed significant moderate to strong correlations (*r* = .419 − .689, all *p* < .001) with the EDE-Q. Weaker but significant associations were found for Negative Attitudes towards Obesity and Muscle Building. Only males showed a significant correlation with Muscle Building, and only females did so for Excessive Exercise. Correlations were largely similar between genders except that in females the EDEQ appeared to correlate more strongly with Cognitive Restraint and more weakly with Muscle Building, although neither difference was significant.

**Table 2:** Correlations in females and males, respectively, between EPSI scales and the EDE-Q Global scale, and z-test for significance of the gender difference between correlations

EPSI scale	EDE-Q Global score		z (*p*) for gender *r* difference
	Females *n* = 184	Males *n* = 163	
Body Dissatisfaction	0.689*	0.674*	0.258 (0.398)
Binge Eating	0.438*	0.439*	− 0.011 (0.495)
Cognitive Restraint	0.666*	0.464*	2.775 (0.003)
Purging	0.510*	0.553*	− 0.553 (0.290)
Restricting	0.506*	0.419*	1.022 (0.153)
Excessive Exercise	0.345*	0.215	1.303 (0.096)
Negative Attitudes toward Obesity	0.323*	0.321*	0.021 (0.492)
Muscle Building	0.167	0.379*	-2.122 (0.017)

Note. *p < .001; EPSI = Eating Pathology Symptoms Inventory; EDE-Q = Eating Disorders Examination Questionnaire.

### Discriminant validity

Most of the EPSI scales correlated significantly (weakly to strongly) with the RCADS (Table 3) in both genders, and there were no significant gender differences in the strength of these associations. To evaluate discriminant validity, the last two columns in Table 3 show z tests of the difference between EPSI-RCADS and EPSI-EDE-Q correlations (from Table 2) separated by gender. In both genders, only one scale showed clear evidence of discriminant validity in that the association between the EDE-Q Global score and Cognitive Restraint was significantly stronger than that between the RCADS and the same scale. In females, Body Dissatisfaction and Purging were near-significantly more strongly associated with the EDEQ than with the RCADS.

**Table 3:** Correlations in females and males between EPSI scales and the RCADS Total Internalizing scale, z-test for significance of the gender difference between correlations, and z-test for differences between EPSI-RCADS and EPSI-EDE-Q correlations in both genders. Significant tests in **bold*** for ease of interpretation*

EPSI scale	RCADS Total Internalizing scale		z (*p*) for gender *r* difference	z (*p*) for difference compared to the EPSI-EDE-Q correlation (Table 3)	
	Females *n* = 184	Males *n* = 163		Females *n* = 184	Males *n* = 163
Body Dissatisfaction	0.525*	0.565*	− 0.525 (0.299)	3.085 (0.002)	1.943 (0.052)
Binge Eating	0.365*	0.442*	− 0.848 (0.198)	1.115 (0.264)	− 0.044 (0.965)
Cognitive Restraint	0.354*	0.235	1.203 (0.114)	**5.386 (< 0.001)**	**3.225 (0.001)**
Purging	0.334**	0.466*	-1.453 (0.073)	2.746 (0.006)	1.357 (0.175)
Restricting	0.444**	0.510*	− 0.788 (0.215)	0.998 (0.318)	-1.365 (0.172)
Excessive Exercise	0.235	0.176	0.568 (0.285)	1.589 (0.112)	0.511 (0.609)
Negative Attitudes toward Obesity	0.195	0.254	− 0.573 (0.283)	1.829 (0.067)	0.906 (0.365)
Muscle Building^1^	0.179	0.342*	-1.616 (0.053)	− 0.167 (0.868)	0.517 (0.605)

Note. *p < .001; EPSI = Eating Pathology Symptoms Inventory; EDE-Q = Eating Disorders Examination Questionnaire; RCADS = Revised Child Anxiety and Depression Scale

### Gender-specific eating pathology

Table 1 shows that females scored higher scores on EPSI Body Dissatisfaction, Cognitive Restraint, and Restricting, as well as the EDE-Q Global scale, with the largest differences found in Body Dissatisfaction and the EDE-Q. Males on the other hand scored moderately more Negative Attitudes toward Obesity and Muscle building on the EPSI, while there were no significant gender differences on Binge Eating, Purging, or Excessive Exercise. Females scored higher on all RCADS subscales except Obsessive-Compulsive.

## Discussion

The aims of the present study was to study gender-specific manifestations of eating pathology in Swedish high-school students as well as to validate a Swedish version of the EPSI [11] by analyzing its factor structure using CFA and assessing convergent and discriminant validity.

We found acceptable CFA estimates and thus support for the proposed 8-factor structure of the EPSI in this convenience sample of high school students. Further, we found support for convergent validity for the majority of the scales, but not for Muscle Building in females or Excessive Exercise in males. Associations with the RCADS were often strong, and comparable to associations with the EDE-Q, suggesting poorer discriminant validity except for Cognitive Restraint in both genders, and with a trend toward discrimination in Body Dissatisfaction and Purging for females. However, ED symptoms are associated with a range of psychiatric symptoms [29], and the “classic” ED symptom scales Body Dissatisfaction and Restricting correlated most strongly with the RCADS, whereas Excessive Exercise, Negative Attitudes toward Obesity, and Muscle Building were weaker. This pattern suggests some specificity of the scales and may be seen to underscore the relevance of concerns around the body and eating for self-esteem and self-image in adolescents [30]. However, the overall limited discriminant validity may suggest caution in interpreting single scales as evidence of ED pathology. Perhaps some EPSI scales may validly function as ED symptom scales in non-clinical ED samples primarily in individuals who have, e.g., high Body Dissatisfaction or Cognitive Restraint. That is, interpretation of for example Muscle Building or Negative Attitudes toward Obesity may indicate those types of ED concerns only in the presence of other ED symptoms, but in the absence of general ED pathology may tap constructs not related to ED. To add important context, previous research concluding support for discriminant validity of the EPSI examined correlation patterns without criteria for what constitutes evidence of “stronger” vs. “weaker” associations with purportedly convergent vs. discriminant constructs (e.g. 5, 6, 11). While our approach found little such support, few measures have been examined in this more stringent way (including those that the EPSI sought to complement, e.g., the EDE-Q), which should be borne in mind when interpreting our findings.

Within-EPSI correlations separated by gender (Table S1) lends support to previous findings of muscularity being more central for EDs in males, as Muscle Building correlated notably more strongly with other symptoms in males than in females. Similarly, Table S2 shows that while the correlation pattern is largely similar between genders, Muscle Building was associated with EDE-Q subscales primarily in males, supporting the relevance of that scale for them specifically, while Excessive Exercise was not strongly associated with eating/shape/weight concerns in males, but more so in females. There were also a gender specific correlation between Muscle building and the RCADS Total Internalizing scale. The correlation was significant only for males and might indicate that muscle building, or execcive focus on own muscularity, is a more general response to psychological distress and not nescessarily a ED symptom. Evidence for such an association has been prensented in a study on male bodybuilders [31]. In their study, frustrated basic psychological needs were associated with drive for muscularity and muscle dysmorphia.

Gender differences in EPSI mean scores were fairly consistent with a previous gender norm study on U.S. college students [11], and while our high school students appeared to score about one *SD* lower in both genders compared to another U.S. study [6]. However, that study scored the EPSI 1–5 rather than 0–4 as in other publications (personal communication, Kelsie T. Forbush, Nov 28, 2023), and simply adding 1 for each constituent item in the EPSI scales to our means brought them overall in line with their findings. Our mean scores were slightly higher on most scales than in a Chinese-speaking U.S. sample [4]. Concerning the other measures, females scored higher on the EDE-Q and all RCADS subscales, consistent with previous findings [32, 33], except RCADS Obsessive-compulsive, where scores were nearly identifal across genders. Whether this is due to the Swedish translation or other factors is unclear, although a Dutch and a Danish study both found only a less-than-small effect size for gender differences among children and adolescents on this subscale [33, 34], whereas effects for the other subscales were generally laerger, and a Norwegian study found that the smallest gender effect obtained for that subscale [32].

### Strengths and limitations

Study strengths include that the sample included both females and males, so that potential gender differences could be examined. Another strength is that the sample consisted of adolescents, as previous studies have mainly been in adults. Most EDs emerge in adolescence [35] and finding instruments that can capture a wide range of initial ED symptoms is therefore valuable.

There are however several limitations including the use of a convenience sample which limits generalizability of the results to adolescents in general. Further, we could not evaluate reasons for external attrition (non-participation), which may have inserted potential bias to the results. In addition, 17 students (4.7%) were over 18 years of age, and the RCADS was designed for ages up to 18. However, one study found good psychometric properties in an RCADS version adapted for adults [36], where the adaptations concerned items about being away from parents or worry about school situations, adaptions which would arguably not be necessary to our high school sample. Also, the RCADS has no specified time frame whereas both the EDEQ and the EPSI asks about the last four weeks, which suggests caution when comparing discriminant and convergent validity since shared method variance might increase convergent but decrease discriminant associations. However, the limited discriminant validity we found argues against this having biased our findings in favor of the EPSI. We did not examine gender-based measurement invariance in this study, suggesting caution when comaring males and females, but as noted, previous studies have found evidence for invariance in other language versions [11, 16, 18]. Lastly, as this along with the majority of previous studies was a non-clinical sample, the factor structure of EPSI in clinical samples remains rather unexplored.

## Conclusions

The Swedish version of the EPSI is a psychometricly sound measure of eating pathology. The eight-factor version enables detailed assessment of different domains of ED symptoms. EPSI includes symptom domains that are not covered in most widely used self-report measures and is thus perhaps better at capturing male specific ED pathology, specifically muscularity-oriented cognitions and behaviours. However, the overall poor discriminant validity in our study may suggest caution in interpreting single scales as evidence of ED pathology.

### Electronic supplementary material

Below is the link to the electronic supplementary material.

Supplementary Material 1

## Data Availability

The datasets used and/or analysed during the current study are available from the corresponding author on reasonable request.

## Footnotes

1 From “Development and validation of the Eating Pathology Symptoms Inventory,” by Forbush, K. T, et al. 2013, Psychological Assessment, 25, 859–878. Copyright © 2011 by Kelsie T. Forbush. Reproduced with permission. No further reproduction, modification, or distribution of the Eating Pathology Symptoms Inventory, derivative versions, or translated versions is permitted without advance, written permission from the copyright holder (Dr. Kelsie Forbush).
