# Supplementary material for: Validation of the eating pathology symptoms inventory (EPSI) in Swedish adolescents
Source: J Eat Disord. 2024 May 27;12:68. doi: 10.1186/s40337-024-01027-7 (PMC11129359; doi:10.1186/s40337-024-01027-7)
Supplement: Supplementary file 1 — Supplementary Material 1 [file 40337_2024_1027_MOESM1_ESM.docx]

Table S1. *Correlations between EPSI subscales by gender.*

| EPSI subscale | Body Dissatisfaction | | Binge Eating | | Cognitive Restraint | | Purging | | Restricting | | Excessive Exercise | | Negative Attitudes toward Obesity | | |
| --- | --- | --- | --- | --- | --- | --- | --- | --- | --- | --- | --- | --- | --- | --- | --- |
|  | Males | Females | Males | Females | Males | Females | Males | Females | Males | Females | Males | Females | Males | Females |  |
| Binge Eating | 45** | .45** |  |  |  |  |  |  |  |  |  |  |  |  |  |
| Cognitive Restraint | .34** | .56** | .39** | .28** |  |  |  |  |  |  |  |  |  |  |  |
| Purging | .47** | .35** | .41** | .36** | .37** | .53** |  |  |  |  |  |  |  |  |  |
| Restricting | .43** | .47** | .37** | .26** | .32** | .46** | .56** | .49** |  |  |  |  |  |  |  |
| Excessive Exercise | .19* | .28** | .44** | .12 | .49** | .49** | .17* | .31** | .23** | .26** |  |  |  |  |  |
| Negative Attitudes toward Obesity | .33** | .26** | .37** | .28** | .22** | .40** | .27** | .46** | .24** | .28** | .25** | .32** |  |  |  |
| Muscle Building | .36** | .21** | .53** | .30** | .41** | .20** | .41** | .35** | .38** | .24** | .57** | .39** | .32** | .23** |  |

** *p*<.01, **p*<.05

Note: EPSI=Eating Pathology Symptoms Inventory

Table S2. *Correlations between EPSI and EDE-Q by gender.*

|  | EDE-Q scale | |  | |  | |  | |  | |
| --- | --- | --- | --- | --- | --- | --- | --- | --- | --- | --- |
| EPSI subscale | Restraint | | Eating Concern | | Shape Concern | | Weight Concern | | Global Score | |
|  | Males | Females | Males | Females | Males | Females | Males | Females | Males | Females |
| Body Dissatisfaction | .34** | .52** | .57** | .52** | .70** | .75** | .61** | .69** | .67** | .69** |
| Binge Eating | .34** | .28** | .46** | .53** | .38** | .39** | .31** | .41** | .44** | .44** |
| Cognitive Restraint | .62** | .69** | .33** | .57** | .26** | .58** | .35** | .58** | .46** | .67** |
| Purging | .45** | .51** | .61** | .52** | .42** | .39** | .42** | .45** | .55** | .51** |
| Restricting | .36** | .49** | .39** | .41** | .32** | .46** | .35** | .48** | .42** | .51** |
| Excessive Exercise | .42** | .33** | .11 | .34** | .04 | .30** | .16* | .29** | .22** | .35** |
| Negative Attitudes toward Obesity | .21** | .32** | .22** | .32** | .29** | .26** | .34** | .28** | .32** | .32** |
| Muscle Building | .43** | .13 | .31** | .17* | .23** | .12 | .31** | .19** | .38** | .17* |

** *p*<.01, **p*<.05

Note: EPSI=Eating Pathology Symptoms Inventory
